# Blocking and its Response to Climate Change

**DOI:** 10.1007/s40641-018-0108-z

**Published:** 2018-07-20

**Authors:** Tim Woollings, David Barriopedro, John Methven, Seok-Woo Son, Olivia Martius, Ben Harvey, Jana Sillmann, Anthony R. Lupo, Sonia Seneviratne

**Affiliations:** 1 Department of Physics, Atmospheric, Oceanic and Planetary Physics, University of Oxford, Parks Rd, Oxford, OX1 3PU UK; 2 Instituto de Geociencias (IGEO), Consejo Superior de Investigaciones Científicas - Universidad Complutense de Madrid (CSIC-UCM), Madrid, Spain; 3 Department of Meteorology, University of Reading, Reading, UK; 4 School of Earth and Environmental Sciences, Seoul National University, Gwanak-ro 1, Gwansk-gu, Seoul, South Korea; 5 Institute of Geography, Oeschger Centre for Climate Change Research, University of Bern, Bern, Switzerland; 6 National Centre for Atmospheric Science, University of Reading, Reading, UK; 7 Center for International Climate Research (CICERO), Gaustadalleen 21, 0349 Oslo, Norway; 8 Atmospheric Science Program, School of Natural Resources, University of Missouri, Columbia, MO USA; 9 Institute for Atmospheric and Climate Science, ETH Zürich, Zurich, Switzerland

**Keywords:** Atmospheric dynamics, Extreme events, Storm tracks

## Abstract

**Purpose of Review:**

Atmospheric blocking events represent some of the most high-impact weather patterns in the mid-latitudes, yet they have often been a cause for concern in future climate projections. There has been low confidence in predicted future changes in blocking, despite relatively good agreement between climate models on a decline in blocking. This is due to the lack of a comprehensive theory of blocking and a pervasive underestimation of blocking occurrence by models. This paper reviews the state of knowledge regarding blocking under climate change, with the aim of providing an overview for those working in related fields.

**Recent Findings:**

Several avenues have been identified by which blocking can be improved in numerical models, though a fully reliable simulation remains elusive (at least, beyond a few days lead time). Models are therefore starting to provide some useful information on how blocking and its impacts may change in the future, although deeper understanding of the processes at play will be needed to increase confidence in model projections. There are still major uncertainties regarding the processes most important to the onset, maintenance and decay of blocking and advances in our understanding of atmospheric dynamics, for example in the role of diabatic processes, continue to inform the modelling and prediction efforts.

**Summary:**

The term ‘blocking’ covers a diverse array of synoptic patterns, and hence a bewildering range of indices has been developed to identify events. Results are hence not considered fully trustworthy until they have been found using several different methods. Examples of such robust results are the underestimation of blocking by models, and an overall decline in future occurrence, albeit with a complex regional and seasonal variation. In contrast, hemispheric trends in blocking over the recent historical period are not supported by different methods, and natural variability will likely dominate regional variations over the next few decades.

## Introduction

The term ‘blocking’ refers to a class of weather systems in the middle to high latitudes. While many meteorologists would agree on whether a particular feature constitutes a cyclone, for example, there is regular disagreement over what should actually be considered a block. Common characteristics are persistence, quasi-stationarity and obstruction of the usual westerly flow and/or storm tracks. Blocks often, but not always, exhibit a large anticyclonic anomaly and reverse the zonal flow such that net easterly winds are seen in some part of the blocked region. By disrupting the usual westerly flow for an extended period such as a week or even longer, these events are often associated with regional extreme weather, from heatwaves in summer to severe cold in winter.

**Fig. 1:**
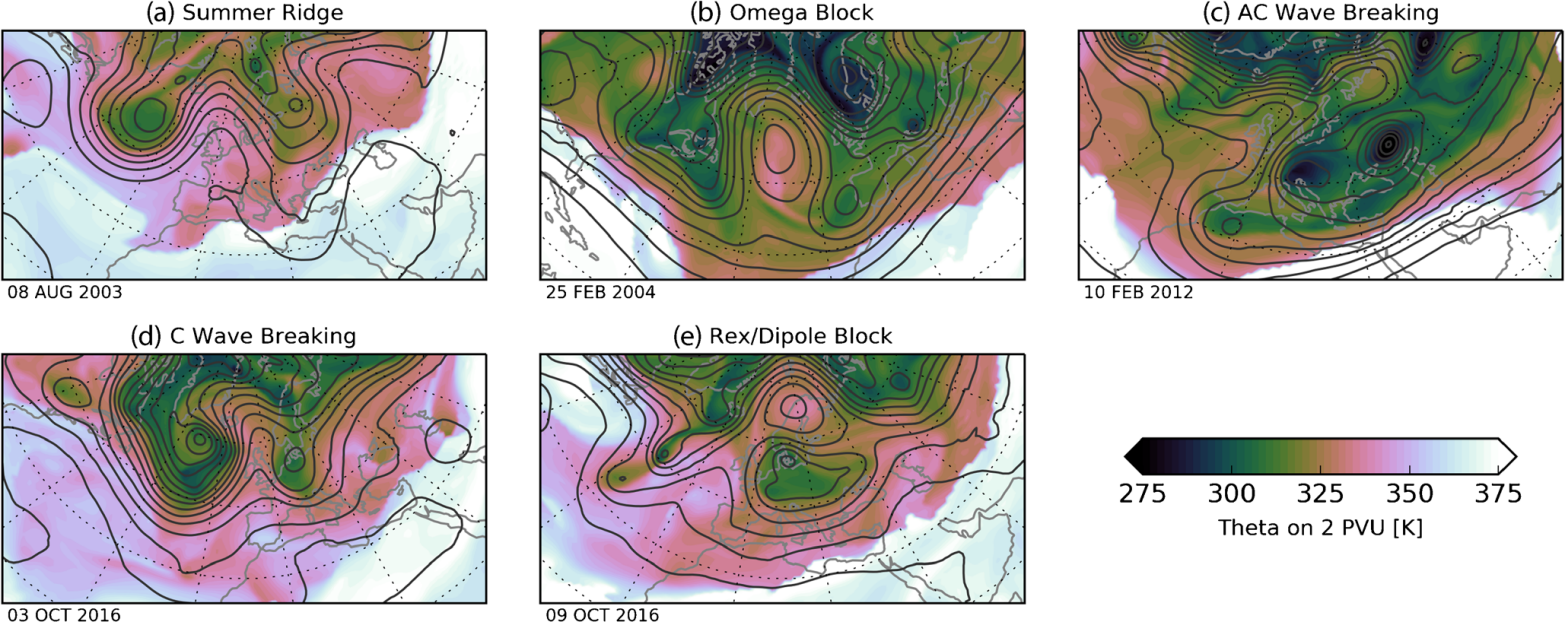
Example North Atlantic blocks. Snapshots of (colour shading) potential temperature θ on the dynamical tropopause (PV = 2 PVU) and (contour lines) geopotential height at 500 hPa (contour spacing 60 m) for the dates indicated. Data is from ERA-Interim

Some examples are shown in Fig. 1 to provide an indication of the range of circulation patterns that have been referred to as blocking. The simplest, but also perhaps the most contentious in terms of blocking definition, is a stationary ridge in a large-amplitude Rossby wave. In these, low potential vorticity (PV), air is advected from the subtropics and is therefore anomalously anticyclonic relative to its surroundings [1], with stationarity being achieved if the Rossby wave has a near zero phase speed. The ‘omega block’ is similar but the poleward diversion is even larger in amplitude with some closed contours in geostrophic stream function (termed a meridional block by [2]). The other configurations shown involve Rossby wave breaking, where the extended ridge is folded over in either a cyclonic or anticyclonic sense [3, 4]. In the wave-breaking events, typically the meridional PV gradient is reversed, and the PV anomalies can form a ‘dipole block’ with the anticyclonic PV anomaly on the poleward side of the cyclonic anomaly. This situation is often described as the ‘Rex Block’ following Rex [5] although it was also pictured in Berggren et al. [6].

This range in blocking patterns is accompanied by a variety of impacts and dynamical mechanisms. As a result, a large number of blocking indices have been proposed in the literature, as different groups present their own particular interpretations and definitions of blocking. This wide range of methods can be confusing to researchers in different fields, but it at least partly reflects the diversity in blocking systems themselves. Given this diversity, most confidence can be placed on results which are supported by several different methods. For example, many studies using different methods have identified blocking as a sporadic and hence highly variable phenomenon, with large fluctuations from seasonal to decadal time scales [7, 8]. Some of this variability may be forced from the tropics [9–11], or by mid-latitude sea surface temperatures [12–14], while some can arise purely from local mid-latitude dynamics (e.g. [15]).

Blocking has often been a cause for concern in association with climate change. This is partly because blocking frequency has generally been simulated poorly by climate models, and even by numerical weather prediction models in medium-range forecasts [16]. However, the lack of a complete dynamical theory for blocking means that we are reliant on these imperfect numerical models for predictions of future blocking behaviour.

This short review does not attempt to provide a complete overview of all aspects of blocking, but is instead focused on the projected changes in blocking occurrence and characteristics under climate change scenarios and the factors which directly relate to these. While most of the literature concerns Northern Hemisphere blocking, many of the concepts reviewed relate equally well to Southern Hemisphere blocking. We begin with a very brief introduction to the dynamics involved in blocking life cycles and the associated impacts. We then give an overview of the methods used to identify blocking in gridded datasets. From there we turn to the climate models, firstly to assess their skill in representing blocking and secondly to summarise the projected changes for blocking and what we know about the underlying physics.

## Dynamics of Blocking

A major weakness in dynamical meteorology is that there is currently no comprehensive theory capturing the different processes acting at all stages of the blocking life cycle: onset, maintenance and decay.

A key characteristic of block onset is a rapid poleward displacement of subtropical air, setting up a large-scale extended ridge, within a Rossby wave pattern on the mid-latitude jet stream. Since both potential temperature (θ) and potential vorticity (PV) are approximately conserved following air parcels, we can identify the air within the extended ridge with a low PV anomaly on a surface of constant θ, or as a high θ anomaly on the dynamic tropopause (a surface of constant PV) as shown in Fig. 1. The ridge extension typically occurs rapidly, on the timescale of 1–3 days. There is often a wide range of scales at play in blocking onset, involving both planetary-scale waves, which tend to be close to an equivalent barotropic structure in the vertical, and baroclinic waves central to synoptic-scale weather systems. Regarding the former, blocking-like flow configurations can arise from purely planetary-scale dynamics in severely simplified systems (e.g. [17, 18]) due to interactions of the background flow with Rossby waves [19] or from the interaction of Rossby waves of different wavelengths [20, 21]. Observations have frequently shown that such waves can be excited in the tropics and propagate into the mid-latitudes where they interact with the background flow and can contribute to blocking formation [22, 23].

Other authors point to a central role of a rapid cyclogenesis event for the establishment of blocks [24–26]. One important aspect is that the cyclone moves slowly so that air mass trajectories can travel a long way polewards within the warm sector. An excellent example occurred at the beginning of October 2016 where a slow-moving rapidly growing cyclone southwest of Iceland contributed to downstream ridge building (Fig. 1d) the onset of Scandinavian blocking (Fig. 1e). This event was observed in detail by multiple aircraft as part of the North Atlantic Waveguide and Downstream Impacts Experiment (NAWDEX; see Schaefler et al., [27] for more detail on this example and the connection with reduced predictability). However, in observed cases, it is difficult to establish cause and effect since the large-scale ‘steering flow’ approaching the block region before onset is often weak, perhaps due to a change in the planetary-scale conditions, which would slow cyclone propagation and enhance meridional extension. This implies that the synoptic-scale eddies and planetary-scale background flow are often tightly coupled in blocking onset. As an additional complexity, the relative importance of synoptic-scale and planetary-scale forcing varies regionally [28, 29].

An essential aspect of a block is that the dynamics are able to maintain it in the same position relative to an observer on the ground, even though there may be strong westerly flow upstream and downstream. In the simplest case of an extended ridge embedded in a large-amplitude Rossby wave train (e.g. Fig. 1a), this can be understood as a balance between advection by the background zonal flow and westward propagation by the Rossby wave pattern. However, when the large-amplitude disturbance is confined to a limited zonal sector, more complex explanations are required [30].

Some idealised dynamical theories have been proposed for block maintenance, such as modon theory involving self-induced advection of a coherent vortex dipole propagating against a uniform flow [31]. However, blocks are typically surrounded by complex, time-varying flow which could disrupt such a structure. The process most often invoked for block longevity is a positive feedback of synoptic-scale eddies on the blocking structure [20, 32–37]. This typically involves the meridional stretching of the transient eddies due to the diffluent flow upstream of the block, which can encourage further wave breaking and feed vorticity anomalies into the block (e.g. [30, 38]). While blocking patterns appear stationary, the upper-level flow is hence highly dynamic with old anticyclonic air masses being replaced by new ones [1]. The persistent forcing of stationary planetary Rossby waves, for example by anomalous tropical circulations, is also likely to play a role in maintaining blocking in some cases [23, 39].

There is increasing awareness of the importance of diabatic effects in the dynamics of blocking onset and maintenance [40, 41]. Poleward moving air within the warm sector of an intensifying cyclone experiences strong dynamical forcing of ascent and is called the ‘warm conveyor belt’ (WCB). Latent heat release amplifies the large-scale ascent and also tends to amplify cyclone growth rate. Heating enables the WCB air to cross θ-surfaces so that the outflow is at a higher level, enabling transport from the boundary layer to tropopause level in ridges. Madonna et al. [42] showed that the average θ-increase in WCBs is 20–25 K. Following trajectories within a WCB, the PV increases below the heating maximum but then decreases again above the heating, such that the average PV of the outflow is expected to be approximately equal to the PV of the inflow [43]. Therefore, the chief influence of the latent heating, relative to dry dynamics, is to create a greater anticyclonic anomaly because the outflow is at a higher level (where the background PV is higher) rather than because the air mass PV value has decreased. A greater proportion of WCB air also turns anti-cyclonically into the ridge, rather than wrapping cyclonically about the cyclone [44]. The divergent wind component advects the tropopause at the outflow level such that the ridge expands further polewards than it would through the influence of the rotational flow in the Rossby wave alone [45].

Recently, Pfahl et al. [46] quantified the relative contributions of adiabatic and diabatic transport pathways into the low PV anomaly of blocks. Back trajectories showed that 30–45% of the air parcels in the low PV anomaly of blocks experience heating (> 2 K) over the last 3 days, implying that latent heat release is of high importance for ridge building and block maintenance. It is not clear whether cyclones with stronger diabatic heating are more likely to cause block onset through enhanced diabatic mass transport into the ridge, or whether the positive feedback of heating on cyclone growth rate and meridional extension is a greater effect.

The slow decay of blocks has often been linked to radiative decay of anomalies, but the vertical structure of long-wave cooling might actually act to enhance upper tropospheric anticyclonic anomalies [47]. Block decay is more likely associated with a breakdown of the maintenance process or disruption through advection by other systems, so that the low PV anomaly can be reabsorbed into the subtropical low PV pool [48].

Several studies have recently addressed lead-lag connections between winter blocking and polar stratospheric variability. Regional blocking influences the polar vortex strength by modulating the upward propagation of tropospheric planetary waves into the stratosphere. This influence depends on the geographical location of the block, which can lead to warming of the polar stratosphere through mainly constructive interference with the climatological planetary waves (e.g. [49–55]). In addition, several studies have also reported significant increases in high-latitude blocking frequency and/or duration following stratospheric sudden warming events, particularly over the Atlantic [56–58].

## Impacts and Links to Extremes

In the regions where blocking typically occurs, the prevailing oceanic westerly flow and associated winds provide warmth in winter and chill in summer. When these winds are obstructed during blocking, the result is therefore a seasonal extreme: cold in winter and hot in summer. Reduced cloud cover in the anticyclonic regions also gives a similar effect, with net surface warming in summer and cooling in winter. These relationships differ in importance seasonally, with thermal advection associated with easterly or northerly winds dominating in winter, but radiative effects being more important in summer [59, 60–64]. In addition, the temperature responses vary substantially depending on the type and location of the blocking pattern [64]. Blocking events in spring and autumn generally attract less interest as they do not lead to the warmest or coldest days annually, but they can still have strong impacts, for example on the agricultural sector in the all-important growing season [65, 66]. For most of the year, Euro-Atlantic blocks enhance the likelihood of heatwaves beneath the anticyclonic region and cold spells equatorward and downstream of the blocking high [64, 67]. Blocking also has strong hydrological impacts, most obviously with dry conditions in the anticyclonic region contributing to droughts. In contrast, regions adjacent to the block can experience extreme rainfall due to the persistent deflection of synoptic storms along the same path [68]. An extreme and unusual example of this was the steering of Hurricane Sandy westwards by a blocking high over Greenland [69]. The clear sky and air stagnation conditions underneath European blocking highs enhance the concentrations of particulate matter during winter and the photochemical build-up of surface ozone during spring and summer, eventually exceeding the air quality targets of these pollutants over some regions of Europe [70, 71].

The strongest impacts of blocking occur due to its persistence, which can allow temperature and moisture anomalies to build up over one or more weeks. Blocking has hence been a key contributing factor to several notable extreme events, for example the European heatwave of 1976 [72], the extreme heatwave in the southern and southeastern USA in 1980 [73], the Russian heatwave of 2010 [74] and the cold European winter of 2010 [75]. Such events are often extremely persistent to the extent that they stand out as clear outliers from the distribution of event durations. In some of these cases at least, there is evidence of remote driving which supports the block, for example through a forced Rossby wave train from the tropics [39, 76]. Local feedbacks from anomalies in soil moisture can clearly amplify the surface heat during summer blocks [77–79]. It is not clear to what extent this feeds back to influence the blocking circulation pattern itself, although some studies report potential effects [80, 81]. Extreme temperatures in mega-heatwaves have contributions from the combined multi-day memory of the land surface and the atmospheric boundary layer as well as heat advection from neighbouring regions, so that a realistic representation of land–atmosphere interactions in climate models is crucial for simulating extreme heatwaves [82, 83].

## How Is Blocking Measured?

The range of systems interpreted as blocking, as shown in Fig. 1, has led to a diversity of blocking definitions. This makes the comparison across studies not straightforward and raises concerns on the sensitivity of the results. All objective blocking methods determine local and instantaneous blocked conditions on a gridded field using a so-called blocking index. Additional criteria are imposed to ensure that the blocking events have minimum spatial extension, quasi-stationarity and persistence (typically 4 or 5 days). The employed datasets vary in their spatial and temporal resolutions, the meteorological variable (geopotential height or materially conserved PV-based dynamical fields) and the vertical level (500 hPa, the upper troposphere-lower stratosphere or the dynamical tropopause). Although upper tropospheric fields may better capture blocking in all seasons [84], these choices ultimately produce similar blocking climatologies when compared systematically under the same method [85–87].

The largest discrepancies among climatologies result from the blocking index, which can take a range of forms, each highlighting different features of a block. The existing blocking indices can be classified into two broad types, based on absolute and departure fields (see [88] for a review). Traditionally, the former identified mid-latitude blocking highs on a 1-D basis by requesting several meridional gradient criteria around a constant latitude representative of the climatological jet stream (e.g. [89]). Subsequent modifications have been applied to account for spatial [90] or seasonal [85] variations of the jet stream, or to design more optimal filters [91]. Others have extended the approach to 2-D by applying the blocking index within a latitude band, avoiding the need of a reference latitude (e.g. [86, 92]). In doing so, the focus turns to Rossby wave breaking [93], which recognises blocking as a high-low dipole (Fig. 1e), regardless of whether it is dominated by the poleward anticyclone (as in traditional methods) or the equatorward cyclone. This approach captures classical mid-latitude blocks associated with the weakening of the jet stream, but also high-latitude and low-latitude blocks, which rather involve southward and northward shifts of the jet stream respectively [94, 95]. In contrast, anomaly methods search for field departures from the time mean (i.e. anomalies exceeding a given threshold), emphasising the 2-D anticyclonic area of the blocking high (e.g. [96–98]). Recent studies have combined both traditions into a hybrid index of height anomalies associated with meridional reversals in order to reduce discrepancies and minimise ‘misdetections’ arising from the single approaches (e.g. [88, 99]). Other studies have also proposed local wave activity, a dynamical measure of the waviness of the jet stream, as a diagnostic of blocking (e.g. [100, 101]). This approach is able to capture blocking but also other events associated with meridional displacements of PV contours.

**Fig. 2:**
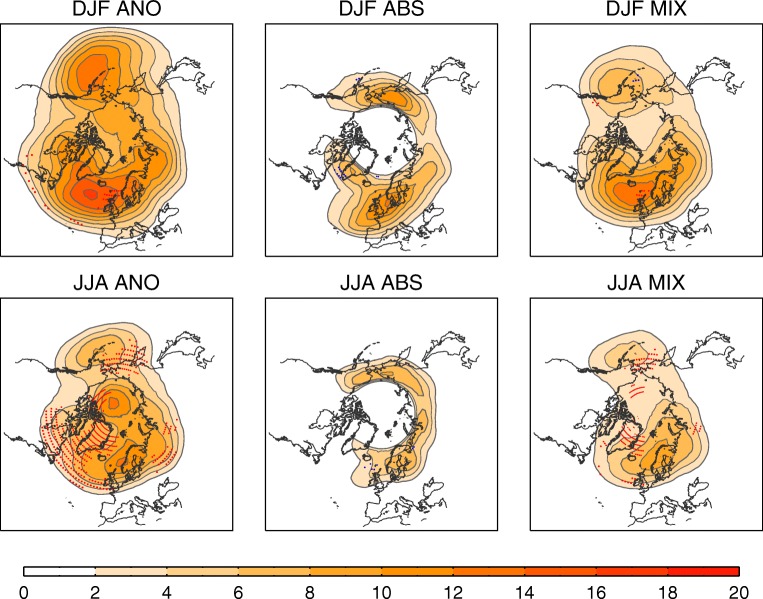
Blocking climatology. (top) Winter and (bottom) summer blocking frequency (percentage of days in the season, with 2% corresponding approximately to 2 blocked days per season) for 1958–2012, using: (left) the anomaly method (ANO), (middle) the absolute method (ABS), and (right) the hybrid method (MIX). Shown is the multi-reanalysis mean based on NCEP/NCAR, ERA-40 + ERA-Interim and JRA-55. Red/blue dots indicate regions with significant (*p* < 0.05, Mann-Kendall test) increasing/decreasing trends in at least two of the three reanalyses

Figure 2 shows winter and summer mean blocking frequency from three different blocking indices spanning the aforementioned approaches, and three different reanalyses. Briefly, these methods all use Z500 and are based on anomalies (ANO), absolute field reversal (ABS) or a combination of the two (MIX); see the Appendix for more details. Climatological features are reasonably robust across reanalyses, and all indices display more frequent blocking in winter than in summer, with a preference for oceanic blocking in winter and continental blocking in summer. However, there are differences in the reported frequency and preferred locations.

Also indicated in Fig. 2 are locations where significant trends are identified in the reanalysis data over the period 1958–2012. While some statistical methods have suggested trends in related atmospheric circulation fields over the satellite period [102], our analysis of conventional blocking indices does not identify robust hemispherical trends over this longer period. Moreover, regional trends in blocking frequency (e.g. the positive summer trend in Greenland blocking, [103]) are also seen to vary depending on the index. One potential risk in identification of trends such as these is that the geopotential height is affected by warming in the column underneath, and so increases in the height do not necessarily imply changes in the statistics of synoptic flow. Previous work has also shown that trends in blocking are not generally robust over longer periods [104], and even the satellite era is still a relatively short period over which to distinguish potential anthropogenic trends from natural multidecadal variability. We conclude, therefore, that clear long-term changes in blocking frequency have not emerged from the internal variability in recent observations.

The different blocking methods have their own strengths and weaknesses and hence we consider all indices equally valid. This diversity should be viewed as an opportunity rather than a limitation, since each approach provides different but complementary aspects of the same phenomenon. In this sense, the exploitation of an array of blocking indices is perhaps wiser than the search for a definitive blocking definition. One may argue that an optimal blocking definition should be founded on the underlying dynamics. Unfortunately, a full dynamical understanding of blocking is still an open issue (see Section ‘Dynamics of Blocking’) and several dynamical processes may be at play in the development of blocking. Instead, we suggest the following recommendations for future blocking methodologies. Firstly, a blocking definition should be inclusive, identifying but differentiating between all types of blocks (i.e. with different structures, in different regions and seasons). In this sense, there remains the challenge of distinguishing among high-low dipoles, omega blocks and even open ridges (Fig. 1). Secondly, the use of thresholds should be kept at a minimum and derived from the input data to accommodate seasonal and long-term variations and allow the applicability of the method to different climate states.

## Representation in Climate Models

**Fig. 3:**
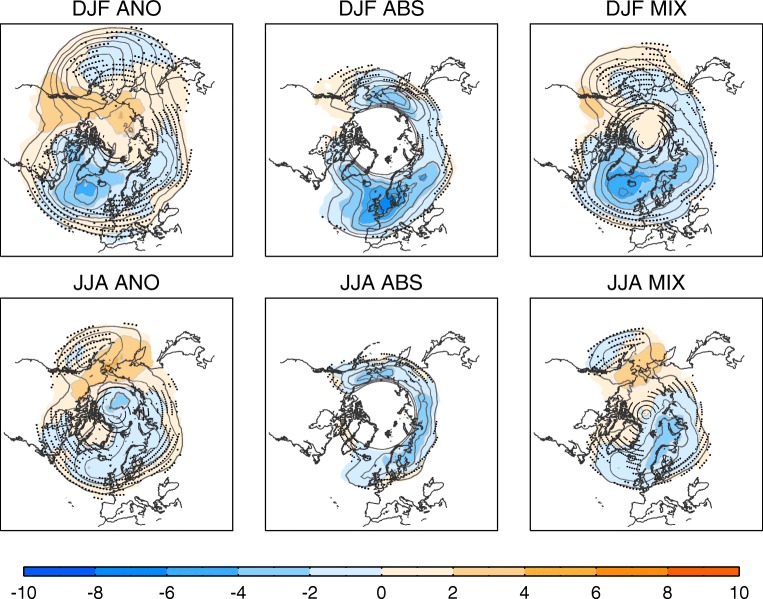
Blocking biases. Multi-model mean (MMM) bias in (top) winter and (bottom) summer blocking frequency (shading, in percentage of days in the season) for the 1961–1990 period: (left) anomaly method (ANO), (middle) absolute method (ABS), (right) hybrid method (MIX). Bias is defined as the difference of blocking frequency climatologies (1961–1990) between the corresponding historical run and the ERA-40 reanalysis. Contour lines depict the ERA-40 blocking frequency climatology (2% intervals starting at 2%). Biases are only displayed over regions with climatological blocking frequencies above 1% in the ERA-40 reanalysis. Black dots denote regions of model disagreement on the sign of the bias (i.e. less than two thirds of the models displaying the same sign). CMIP5 models (one member per model): BNU-ESM, BCC-CSM1-1, BCC-CSM1-1-M, CanESM2, CCSM4, CMCC-CESM, CMCC-CM, CMCC-CMS, CNRM-CM5, FGOALS-g2, FGOALS-s2, GFDL-CM3, GFDL-ESM2M, HadGEM2-CC, HadGEM2-ES*, IPSL-CM5A-LR, IPSL-CM5A-MR, IPSL-CM5B-LR, MIROC5, MIROC-ESM, MIROC-ESM-CHEM, MPI-ESM-LR, MPI-ESM-MR, MRI-CGCM3, NorESM1-M. *The 1981–2005 period is used instead as historical period

Blocking has always presented a challenge for numerical weather and climate models, which tend to underestimate both the occurrence and persistence of events [89, 105, 106]. A recent comprehensive survey found that there has been some systematic improvement over generations of models, particularly in the Pacific sector [106]. However, over Europe, there has been little improvement overall, with only a small number of models now exhibiting blocking frequencies approaching (but still not reaching) observed levels. Figure 3 shows the mean biases from CMIP5 models using the three different blocking indices. This shows a general agreement between methods on an underestimate of Atlantic/European blocking of around 30–50% of the observed frequency in winter and around 10–30% in summer (particularly in the high-latitude Eurasian region). In contrast, biases in the Pacific sector are smaller and often not significant. This highlights the role of different processes acting in Pacific versus European blocks, and it is possible for one to be improved in a model while the other degrades [107, 108].

Anecdotal evidence suggests that blocking frequencies can still be fragile even in relatively capable models, with the gains accomplished in one model version sometimes lost in the following version. Apparent blocking improvements in a model can also occur through compensation of errors [109]. A further problem is that the sporadic nature of the event leads to strong natural variability in blocking frequencies, which can often hamper model evaluation when short periods prone to sampling uncertainty are used for test simulations. Despite these problems, experience has shown that modelled blocking can (sometimes, but certainly not always) be improved by:

1. Increases in horizontal resolution, which improves transient eddy forcing of blocks (Matsueda et al. [16, 107, 108]).
2. Increases in vertical resolution, which enables better representation of tropopause dynamics [110] and perhaps diabatic ascent and outflow from WCBs.
3. Reduction or elimination of SST biases ([111], though note that AMIP versions are not better in many models [106]).
4. Improved orography, which forces enhanced stationary wave patterns [107, 112].
5. Improved physical parameterisations [113], such as of convection [114] and drag [114, 115].
6. Improved accuracy of the dynamical core numerical scheme [116].

While generally encouraging, this list does highlight that there are many ways to achieve bad blocking representation in a model, but not an easy recipe to guarantee good blocking simulation.

Several studies have found that biases in blocking are intimately connected to biases in the mean flow, such that a region with low blocking will typically exhibit a mean westerly wind bias [117, 118]. While seemingly a classic chicken-and-egg problem, analysis indicates that low blocking frequencies alone cannot explain the mean bias, but in converse the mean state bias can, in good models at least, often statistically ‘explain’ the blocking bias [106, 117]. This highlights the sensitivity of many blocking indices to the mean state, so that some diagnosed changes in blocking, either in model biases or responses to forcing, may simply reflect a mean shift of the climate rather than any change in the variability of the flow [119]. However, in some cases, it is clear that biases in blocking are intimately related to structural errors in the representation of jet variability [120].

Despite overall disappointing progress in representing European blocking in a multi-model mean sense, the existence of a small number of models with reasonable blocking behaviour offers considerable opportunity. Some studies have identified a small subset of models with reasonable blocking structures and frequencies (within about 20% of the observed) and exploited these for more targeted investigation [121]. These are also typically the models for which mean state biases can account for much of the remaining underestimate in blocking. Despite the presence of biases, the relationship between extreme temperature and blocking is often captured by models, at least when large ensemble simulations are used [122]. An additional cause for optimism is that blocking biases are slightly weaker in summer than winter for some regions (Fig. 3 and [87]), when the association with heatwaves makes blocking of particular concern in a warming world. Some bias does remain, however [108], and further work would be welcome to investigate this, as many studies continue to focus on winter events only. Insights to reduce biases in climate models could be gained from the experience with prediction models. As a result of recent improvements to forecasting models, impressive skill is evident in probabilistic predictions of blocking and related weather regimes in the medium range [123, 124] and even in some cases on the seasonal time scale [125]. Forecasts can be highly skillful once a blocking is established but predicting the onset of blocking can still be challenging ([27, 126, 127]).

## Projections of Future Climate

**Fig. 4:**
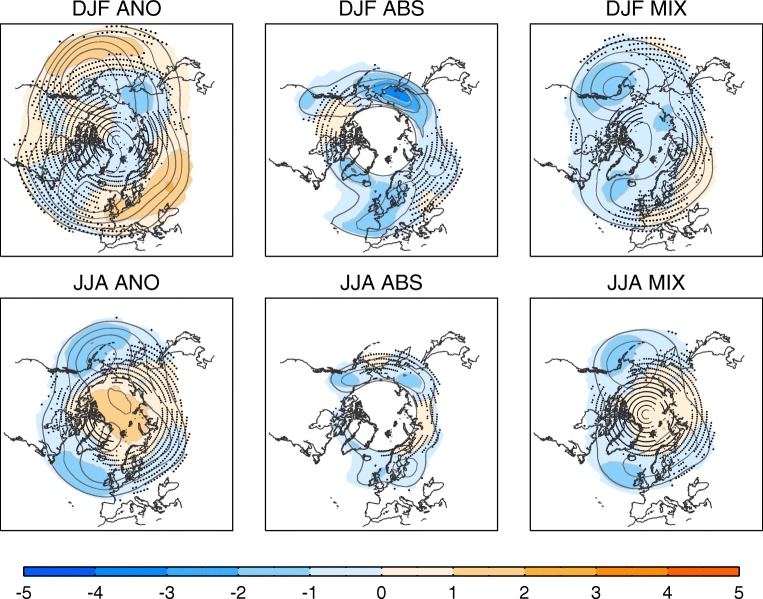
Blocking projections. Multi-model mean (MMM) RCP8.5 projections of (top) winter and (bottom) summer blocking frequency changes (shading, in percentage of days in the season) for the 2061–2090 period with respect to the 1961–1990 period of the historical simulation: (left) anomaly method (ANO), (middle) absolute method (ABS), (right) hybrid method (MIX). Contour lines depict the MMM historical (1961–1990) blocking frequency climatology (2% intervals starting at 2%). Changes are only displayed over regions with historical blocking frequencies above 1% in the MMM. Black dots denote regions of model disagreement on the sign of changes (i.e. less than two thirds of the models displaying the same sign). CMIP5 models (one member per model): BNU-ESM, BCC-CSM1-1, BCC-CSM1-1-M, CanESM2, CCSM4, CMCC-CESM, CMCC-CM, CMCC-CMS, CNRM-CM5, FGOALS-g2, FGOALS-s2, GFDL-CM3, GFDL-ESM2M, HadGEM2-CC, HadGEM2-ES*, IPSL-CM5A-LR, IPSL-CM5A-MR, IPSL-CM5B-LR, MIROC5, MIROC-ESM, MIROC-ESM-CHEM, MPI-ESM-LR, MPI-ESM-MR, MRI-CGCM3, NorESM1-M. *The 1981–2005 period is used instead as historical period

Earlier climate model projections such as CMIP3 consistently featured a general reduction in blocking frequency [85] as the mid-latitude jets strengthened and/or shifted poleward in many regions. The more recent CMIP5 model projections have suggested that the responses of blocking frequency to climate change might be weaker and more complex [87, 128]. The Euro-Atlantic and Pacific winter blocking frequencies are projected to decrease on their western flanks but increase on the eastern flanks, suggesting an eastward shift in blocking activity [87, 129–131]. During summer, poleward shifts in blocking activity are reported, leading to decreases in blocking frequency in mid-latitudes [131] but increases in high latitudes [87]. The Urals are among the few regions where blocking may increase in a future climate, although this is not robust across models, studies and even scenarios [128, 131–133]. The quantitative results, however, are somewhat sensitive to the blocking detection methods, as illustrated using the three methods in Fig. 4. In the multi-model ensemble, the largest decreases are detected during winter in the absolute method, but during summer in the anomaly method, with similar changes in the hybrid index. The spatial distribution of the projected changes also differs, suggesting that different methods reveal different aspects of blocking changes and/or are not equally sensitive to changes in the mean vs changes in variability. In the Southern Hemisphere, blocking frequency is also anticipated to decrease, particularly in the Pacific during austral spring and summer, with a hint of meridional displacements. As in its northern counterpart, seasonal changes are more diverse, with regional but not robust increases across the models [134].

These changes in blocking frequency can be directly related with changes in the mean flow and eddies [119, 128, 132, 135, 136]. The projected shift of the Euro-Atlantic blocking, for instance, is consistent with the strengthened Atlantic jet and the eastward extension of high-frequency eddies [136], although it is difficult to separate the cause from the effect. A similar relationship is also found for the Ural blocking. Such consistency, however, has not been reported over the Pacific, suggesting that multiple and perhaps competing factors are contributing to Pacific blocking changes, in agreement with the larger disparity of model projections in this region. Reduced Pacific winter blocking has been related to a weakened and poleward-shifted Hadley circulation [130] and more El Niño (less La Niña) events [128]. While individual models do not support a link with ENSO changes, ensemble projections with prescribed warming in the equatorial Pacific reveal consistent decreases in winter Pacific blocking for a variety of future ENSO responses [131].

The generally decreased blocking frequency in future climate could result in a reduction in weather and climate extremes. However, changes in the background state or local feedbacks could counteract this reduction, for example increases in surface sensible heat flux associated with enhanced soil moisture drying [79, 137]. There are several examples supporting changing relationships between blocking and its impacts. For instance, the cooling effect of Atlantic winter blocking over Europe is projected to weaken [63, 138] due to a reduced land-sea temperature contrast and thermal advection [139, 140]. However, temperature anomalies associated with European winter blocking could shift northeastward, along with changes in blocking location [121, 141], likely leading to stronger impacts in regions that are less affected in the present climate. Similarly, Okhotsk winter blocking may trigger a higher frequency of cold days over Japan [129] and Ural winter blocking may exert a stronger impact on East Asia [132], as already observed in recent decades [142]. The relationship between temperature and blocking will continue to play an important role in the development of cold spells and heatwaves in all seasons [67]. However, it is crucial to use large ensembles of different climate models, as single realisations do not necessarily capture this relationship [122, 143]. The links between blocking and stratospheric variability also seem to remain under climate change scenarios. However, the region of blocking influence on planetary wave propagation may show an eastward shift in the Euro-Atlantic sector, resulting in a more effective influence of blocking on the polar stratosphere [144].

Major efforts are required to better understand the uncertainty in future blocking projections. To this aim, several studies have explored different approaches. One of them entails the selection of the best performing models in historical simulations based on the assumption that model biases may degrade future projections. However, these models do not always agree on their projections [87]. Moreover, models can often predict similar changes in the future despite disparate model performances in present-day runs. A more reasonable tactic invokes improved process understanding. A process-oriented approach has been particularly implemented in the modelling exercises that isolate the blocking responses to different aspects of the global warming pattern. This suggests that upper-level tropical warming is a key factor driving the reduction in blocking due to its effect of strengthening the zonal winds [139]. The influence of near-surface Arctic warming is more contentious, with some studies suggesting this could increase blocking [145–147] but others suggesting a negligible, or even negative change in blocking occurrence [139]. To better identify the dynamical mechanisms of the projected blocking changes and their impacts on weather and climate extremes, further targeted modelling efforts will be necessary.

## Perspective

The generic term ‘blocking’ covers a wide variety of flow patterns. A plethora of blocking indices have been developed as a result, which can be daunting for researchers in other fields. However, it appears better to recognise the diversity than to be overly prescriptive in attempting to formulate a universal definition. Most confidence can then be placed in results which emerge from the application of several different methods, as in the analyses presented here.

Even when different methods agree, however, confidence is still relatively low in projected blocking changes. One reason for this is the lack of theoretical support, with several different physical mechanisms contributing to blocking, which thus far has prevented the development of a theory for the whole life cycle of the event. Another reason is the continued underestimation of blocking activity by climate models, particularly for the Atlantic/European sector in winter. The reliance of blocking on many aspects of numerical model design means that only a handful of models can be considered to have a reasonable simulation of blocking. This leads to a dilemma over whether to include all models in blocking studies or only a subset of the best models. There is no clear solution to this since, for example, it may turn out that the critical ingredient for future blocking change might not be a model’s present-day skill in blocking but its skill in predicting the response in some remote driver, such as the pattern of SST change.

Considering the century-timescale projections, there remains a general agreement between models on an overall decline in mid-latitude blocking occurrence, at least in the hemispherical mean. Important regional, seasonal and methodological differences are emerging, however, which are yet to be fully understood. Regarding the caveats above, it is important to note that the projected changes are generally smaller in magnitude than the model biases and the decadal variability in blocking frequency. It is not clear how important this is, but obviously a model with very little blocking is severely limited in how it can respond to forcing. In many cases, the projected changes in blocking appear to be relatively ‘passive’ consequences of changes in the atmospheric mean state. It remains possible that improved representation of the many dynamical processes involved may lead to blocking responding in a more ‘active’ way to anthropogenic forcing. More targeted, process-based studies with capable models may help to improve confidence in model projections.

One of the less recognised challenges associated with blocking is its strong natural variability, including, for example, a small number of rare but very persistent and high-impact events. There are several practical consequences of this, for example that long time periods or multiple ensemble members are often needed to obtain good sampling statistics of blocking in data. Given the level of natural variability, it is perhaps not surprising that no fully consistent long-term trends in blocking have yet emerged in observations. Given the importance of natural variability for mid-latitude circulation in general [148], it is likely that this will continue to play a leading role in blocking variations over the coming few decades. Coupled with the relatively gradual future decline of blocking in the ensemble projections, this suggests that blocking is likely to remain a major source of extreme weather during this century. This is especially true in summer given the association with heatwaves. The impact of wintertime blocking on temperature is largely due to thermal advection which is likely to weaken in the future, but in contrast the temperature impacts of summertime blocking may strengthen due to soil moisture feedbacks.

## Appendix

We describe the algorithms and thresholds employed herein to identify blocking. They follow common approaches published elsewhere [88]. For simplicity and coherency, the implementation of thresholds and other methodological considerations have been modified with respect to the original studies. Daily geopotential height fields at 500 hPa (Z500) were linearly interpolated to a common 2.5°×2.5° grid before performing the computations.

### Anomaly method (ANO, hereafter, similar to Schwierz et al. [98] but using Z500 instead)

Daily anomalies of Z500 are computed for each grid point as the difference with respect to the climatological mean daily values of the analysed period. For each day, blocks are detected as 2-D areas of at least 2·10^6^ km^2^ extension with Z500 anomalies above the 90th percentile of the Z500 anomaly distribution over 50°-80°N. The same threshold is applied to all grid points, but it is allowed to change with the calendar month by using three-month centered distributions. Quasi-stationarity and persistence are ensured by imposing a minimum percentage of spatial overlap between the blocked areas of successive days (50%) for at least 5 days.

### Absolute method (ABS, hereafter, similar to Davini et al. [86])

For each longitude λ, the following meridional Z500 gradients are computed to the north and south of a given latitude ϕ:

1 $$ {\displaystyle \begin{array}{l} GHGN\left(\lambda, \phi \right)=\frac{\left(Z\left(\lambda, \phi +\Delta \right)-Z\left(\lambda, \phi \right)\right)}{\Delta}<-10 gpm/{}^{\mathrm{o}}\\ {} GHGS\left(\lambda, \phi \right)=\frac{\left(Z\left(\lambda, \phi \right)-Z\left(\lambda, \phi -\Delta \right)\right)}{\Delta}>0\\ {}{GHGS}_2\left(\lambda, \phi \right)=\frac{\left(Z\left(\lambda, \phi -\Delta \right)-Z\left(\lambda, \phi -2\Delta \right)\right)}{\Delta}<-5 gpm/{}^{\mathrm{o}}\end{array}} $$

where 45° < ϕ < 70° N and ∆ = 15° latitude. GHGN and GHGS_2_ are applied to avoid the detection of cut-off lows and subtropical features (‘low-latitude blocks’). A grid point is blocked if the above meridional gradients averaged over ∆/2 in longitude satisfy eq. (1). 2-D blocked areas are required to have a minimum areal extension of 500,000 km^2^. Finally, blocking patterns occurring during consecutive days are considered a blocking event if there is any overlap between their 2-D blocked areas for at least 5 days.

### Hybrid method (MIX, hereafter, similar to Barriopedro et al. [88])

Following ANO, daily blocks are identified as contiguous 2-D spatial signatures with anomalies above a given threshold (the same as in ANO). Similar to ABS, these areas are also required to be associated with meridional Z500 gradient reversals around a reference latitude (*ϕ*_*c*_), defined for each longitude and calendar month as the latitude with maximum variance in the 5-day high-pass Z500 filtered field. This condition is demanded by computing the difference between ∆-width latitudinal averages of Z500:

2 $$ GHGS\left(\lambda, \phi \right)=\frac{\overline{Z}\left(\lambda, \phi :\phi +\Delta \right)-\overline{Z}\left(\lambda, \phi :\phi -\Delta \right)}{\Delta}>0 $$

where *ϕ*_*c*_(*λ*) − Δ/2 < *ϕ* < *ϕ*_*c*_(*λ*) + Δ/2 and ∆ = 15° latitude. The entire 2-D area is blocked if, for at least one of its longitudes, GHGS averaged over ∆/2 in longitude satisfies eq. (2). Finally, minimum cut-off values are required to the 2-D extension (2∙10^6^ km^2^), the fraction of overlap between successive daily blocks (50%) and the duration (5 days).
